# Dynamic Hybrid Flagellar Motors—Fuel Switch and More

**DOI:** 10.3389/fmicb.2022.863804

**Published:** 2022-04-12

**Authors:** Kai M. Thormann

**Affiliations:** Fachbereich für Chemie und Biologie, Institut für Mikrobiologie und Molekularbiologie, Justus-Liebig-Universität Gießen, Gießen, Germany

**Keywords:** flagellum, swimming, swarming, regulation, bacteria, stator swapping

## Abstract

Flagellar motors are intricate rotating nanomachines that are powered by transmembrane ion gradients. The stator complexes are the powerhouses of the flagellar motor: They convert a transmembrane ion gradient, mainly of H^+^ or Na^+^, into rotation of the helical flagellar filament. They are thus essential for motor function. The number of stators synchronously engaged in the motor is surprisingly dynamic and depends on the load and the environmental concentration of the corresponding coupling ion. Thus, the rotor–stator interactions determine an important part of the properties of the motor. Numerous bacteria have been identified as possessing more than one set of stators, and some species have been demonstrated to use these different stators in various configurations to modify motor functions by dynamic in-flight swapping. Here, we review knowledge of the properties, the functions, and the evolution of these hybrid motors and discuss questions that remain unsolved.

## Introduction: Flagella-Mediated Motility

During the billions of years of their existence, bacteria have evolved a range of different systems that allow them to move actively toward environments that are more favorable and to conquer new habitats (Jarrell and McBride, 2008; Wadhwa and Berg, 2021). Among the different means of locomotion, flagella are highly common. Flagella are long, rotating helical protein fibers (Berg and Anderson, 1973; Silverman and Simon, 1974) that extend from the cell surface and act as propellers to drive the cell efficiently through aqueous habitats and through more-structured environments or across surfaces (Kearns, 2010; Kühn et al., 2017).

**FIGURE 1:**
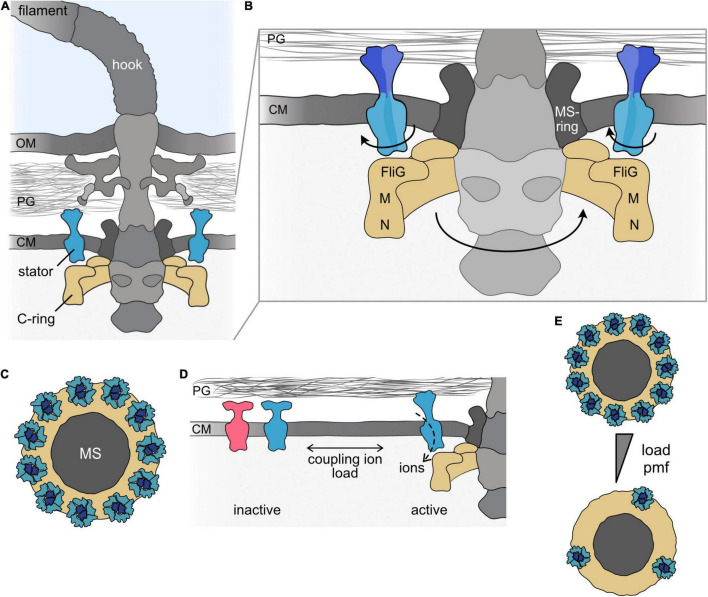
The bacterial flagellar motor. **(A)** Cartoon of the bacterial flagellum of a Gram-negative bacterial cell. The flagellum consists of the long helical filament and the universal joint, the hook, which connects the filament and the flagellar motor. The motor is part of the basal body, which firmly anchors the flagellar apparatus in the cell envelope and, in addition, houses a type III–like protein export system. The main flagellar components involved in torque generation, the rotary C-ring and the stators, are shown in the same colors (here and throughout all figures). **(B)** Magnification of the flagellar motor. In the rotating motor, the ion-conducting stators (here blue) are anchored to the cell wall by a peptidoglycan-binding domain at the C-terminus of the B subunit of the stator. The ion flow leads to rotation of the A-subunits of the stator around the centered B-subunits. The rotation is transferred to the flagellar rotor, the C-ring (depicted in yellow), which consists of multiple copies of the proteins FliG, FliM, and FliN. Rotation of the C-ring is induced by electrostatic interaction of the A-subunits of the stator with FliG. **(C)** Top view on the transmembrane MS-ring (dark gray) to which the C-ring (yellow) is mounted. The stator units are positioned around the MS-ring above the C-ring. In a fully assembled motor of *E. coli*, about 11 stators are engaged with the rotor. **(D)** Stator units are produced as inactive precomplexes, which diffuse in the membrane. Upon engaging with the motor, the stators bind to the peptidoglycan and the ion channel opens. Stator units can also leave the motor to re-join the pool of inactive stator units in the cytoplasmic membrane. The equilibrium is affected by environmental signals, e.g., the concentration of the coupling ion and/or the load acting on the flagellar filament. Stator coupling and uncoupling occurs while the flagellum continues to rotate. Notably, more than one stator type can be present (indicated by the blue and red colors). **(E)** Under conditions of low load or at a low concentration of the coupling ion, only a few stators are engaged with the motor. A single stator is generally sufficient to drive flagellar rotation under conditions of low load. Under conditions of increasing load and/or increasing concentration of the coupling ion (indicated by the gray triangle), the maximal number of stators can be engaged. OM, outer membrane; PG, peptidoglycan; CM, cytoplasmic membrane; M, FliM; N, FliN; MS, MS-ring.

Rotation of the flagellar filament is conferred by a rotating nanomachine, the flagellar motor, to which the filament is connected by a structure that serves as a universal joint structure, the flagellar hook (Figure 1A). Along with the flagellar protein export system, the motor is embedded into the cell envelope to form the flagellar basal body. Motor rotation is powered by transmembrane gradients, most commonly of protons (H^+^) or sodium ions (Na^+^) (Manson et al., 1977; Matsura et al., 1977; Hirota et al., 1981; Li et al., 2011). The majority of flagellar motors are bi-directional and can rotate in both the clockwise and counterclockwise directions, which allows the cells to navigate within environmental signal gradients *via* chemotaxis systems coupled to the flagella. Decades of work by many research groups in concert with constantly advancing techniques, e.g., in fluorescence microscopy and cryo-tomography, have provided deep insights into the structure of the flagellum, the composition of its motor, and the mechanism of rotation (Berg, 2003; Sowa and Berry, 2008; Johnson et al., 2021; Tan et al., 2021; Wadhwa and Berg, 2021).

Active motility provides a huge advantage for many organisms in nature (Fenchel, 2002; Reichenbach et al., 2007; Gude et al., 2020). This benefit, however, always comes with the cost of a substantial investment of energy and resources. For bacteria, not only the assembly but also the operation up to many flagella is a significant metabolic burden, as the cells have to constantly maintain the appropriate ion gradient to power the rotation. In addition, most bacteria thrive in rapidly changing environments and thus have to adjust the performance of their flagella accordingly. To this end, a range of mechanisms has evolved, which include direct binding of effector proteins that act as brakes, accelerators, clutches, or mediators of directional switching, sometimes even including the shedding the flagellar filament and part of the basal body (Minamino et al., 2019; Subramanian and Kearns, 2019; Zhu and Gao, 2020). These mechanisms will not be considered in detail here. Instead, we will review and discuss the inherent properties of the structure of the flagellar motor and its components that allow specific control and adaptation of the performance of the motor.

## A Rotary Machine Driven by Rotary Machines—Rotor–Stator Interaction in the Flagellar Motor

In general, rotational motors require two parts to generate torque between them: a part that rotates, the rotor, and a part that remains static, the stator. In all bacterial flagellar motors, the rotor comprises the cytoplasmic C-ring, which consists of dozens of copies of the proteins FliG, FliM, and FliN (or FliY in some bacterial motors) (Johnson et al., 2021; Tan et al., 2021; Wadhwa and Berg, 2021). The C-ring is shaped like a shallow, inverted cup with a diameter of about 45 nm (in *Salmonella* or *Vibrio*), where FliG is located close to the membrane and FliN is at the distal position within the cytoplasm (Francis et al., 1994; Figures 1A,B). FliG associates with FliF, the protein that forms the MS ring in the stator, which transduces rotation to the flagellar rod.

The stator is formed by distinct, ion-specific stator units. Each stator unit is an independent ion-conducting complex (Braun and Blair, 2001) built from seven copies of two transmembrane proteins, commonly referred to as MotA and MotB (as in the H^+^-dependent motors of *E. coli* or *S. enterica*) (Silverman et al., 1976; Dean et al., 1984; Stader et al., 1986) or PomA and PomB (as in their Na^+^-dependent counterparts in species of *Vibrio* or *Shewanella*) (Asai et al., 1997). MotA (or PomA) is embedded in the cytoplasmic membrane by four transmembrane regions and has a large cytoplasmic loop connecting transmembrane helices two and three and another cytoplasmic extension at the C-terminal end (Zhou et al., 1995). MotB possesses a short N-terminal cytoplasmic segment followed by a single transmembrane helix and a large periplasmic region (Chun and Parkinson, 1988), which harbors a peptidoglycan-binding domain (PBD) at its C-terminal end (De Mot and Vanderleyden, 1994). Each stator unit consists of five MotA/PomA subunits that are arranged in a ring around two MotB/PomB proteins (Deme et al., 2020; Santiveri et al., 2020; Hu et al., 2021). In a fully assembled motor of *E. coli* or *Vibrio* sp., about a dozen stators surround the MS-ring in the membrane, where they are positioned above FliG in the cytoplasmic C-ring and are bound to the cell wall by the MotB/PomB PBD domain (Khan et al., 1988; Reid et al., 2006; Thomas et al., 2006; Beeby et al., 2016; Figures 1B,C). The most recent model predicts that, driven by ion flow through each stator unit, the MotA proteins rotate around the two center MotB proteins and thus are themselves rotary nanomachines. The MotA cytoplasmic sections interact electrostatically with the C-terminal domains of the FliG proteins in the C-ring below the stators (Zhou and Blair, 1997; Zhou et al., 1998; Terashima et al., 2021; Figure 1B). Conformational changes in the C-ring, e.g., by binding of the phosphorylated form of the chemotaxis response regulator CheY, alter the interaction of MotA/PomA with FliG and switch the C-ring‘s rotational direction (Paul et al., 2011; Stock et al., 2012; Santiveri et al., 2020; Hu et al., 2021).

## Dynamics of Rotor–Stator Interaction and Motor Remodeling

As described above, the stator units are the powerhouses of the flagellar motor and determine the nature of the coupling ion and the torque that can be produced. Despite this designation and their role in torque production, the rotor–stator configuration has been demonstrated to be surprisingly dynamic (Armitage and Berry, 2020).

The coupling of stator units to, and uncoupling from, the motors was observed by an incremental increase (or decrease) in torque (Block and Berg, 1984; Blair and Berg, 1988; Muramoto et al., 1994; Sowa et al., 2005) and, later, more directly by microscopic observation of fluorescently labeled stator units (Leake et al., 2006). When they are first assembled, the stator units diffuse as a pool of inactive precomplexes [about 300 in *E. coli* (Leake et al., 2006)] within the cytoplasmic membrane (Figure 1D). At that stage, the periplasmic section of the B-subunit is not bound to the cell wall; it assumes a conformation that prevents premature ion flow through the membrane. Only when they engage with the flagellar motor are the stators activated through conformational changes that allow binding to the peptidoglycan and release of ion flow (Van Way et al., 2000; Hosking et al., 2006; Kojima et al., 2009, 2018; Morimoto et al., 2010; Zhu et al., 2014). Each stator unit can also disengage from the flagellar motor and join the pool of membrane-diffusing inactive precomplexes. Thus, the stators in the flagellar motor are constantly turned over. Notably, stator exchange occurs while the motor continues to function.

The stability of rotor–stator interactions depends on at least two major factors: the ion motive force and the load acting on the filament (Figure 1D). Collapsing the ion motive force results in physical uncoupling and diffusion of the stator units from the motor, e.g., in *E. coli* (Figure 1E), whereas restoring the proton motive force leads to re-incorporation of stators into the motor (Fung and Berg, 1995; Tipping et al., 2013b). A similar behavior occurs in the Na^+^-dependent motor of *Vibrio alginolyticus*: The stators disengage from the motor when Na^+^ in the environment is depleted or when Na^+^ transport through the stator unit is blocked (Fukuoka et al., 2009; Kojima, 2015). The stability of rotor interactions with the stator may be regulated by conformational changes induced by binding of the coupling ion to the stator complex, (Kojima, 2015; Terahara et al., 2017a).

In addition to the concentration of the coupling ion, the load on the flagellum affects the stability of rotor–stator interactions and, by this, the number of stators engaged in the motor. Several studies provide evidence that the number of stators synchronously engaged in the motor increases with progressively rising load on the flagellum and, *vice versa*, decreases when the torque 141 is lowered (Lele et al., 2013; Tipping et al., 2013a; Nord et al., 2017a,b; Wadhwa et al., 2019). The observations support a catch-bond model, where the interaction between the stator and the cell wall is strengthened with increasing force and is lowered with decreasing force (Nord et al., 2017a; Nirody et al., 2019). Thus, at very low load, only one or a few stators drive motor rotation, and engagement of the maximum number of stators occurs only at maximal load or when the rotation is stalled.

The dynamic assembly of the stator ring may save energy by restricting the ion flow through the flagellar motor when the load on the filament is low so that little advantage in propulsion would be gained by using more stators (Armitage and Berry, 2020). The mechanosensing property of the flagellar motor could also allow the cell to determine the viscosity of the environments or attachment to a solid substratum and to elicit an appropriate response (Chawla et al., 2020; Laventie and Jenal, 2020). However, as stator dynamics have been properly determined for only few species, it should be noted that not all bacteria may exhibit stator exchange, e.g., the stator ring of Spirochetes appears to be rather stable (Chang et al., 2019). On the other hand, a number of species have taken this mode of motor regulation one step further, as they can remodel the configuration and performance of the motor using different types of stators.

## The Dynamic Hybrid-Fueled Motors of *Shewanella oneidensis* and *Bacillus subtilis*

The majority of the bacteria that swim by flagellar motility encode a single specific stator unit to drive the corresponding flagellar motor. In contrast, there are a number of species that harbor genes encoding more than one distinct stator unit, among them species of the genus *Pseudomonas*, *Bacillus*, and *Shewanella* (Doyle et al., 2004; Ito et al., 2004, 2005; Toutain et al., 2005; Paulick et al., 2009; Thormann and Paulick, 2010). Some bacteria may use only one of the stators at a time by producing each of the stator units only under certain conditions. Alternatively, the bacteria could be able to use both stators synchronously. In two species, *Shewanella oneidensis* and *Bacillus subtilis*, it has been shown that the flagellar motor allows mixed stator configurations.

**FIGURE 2:**
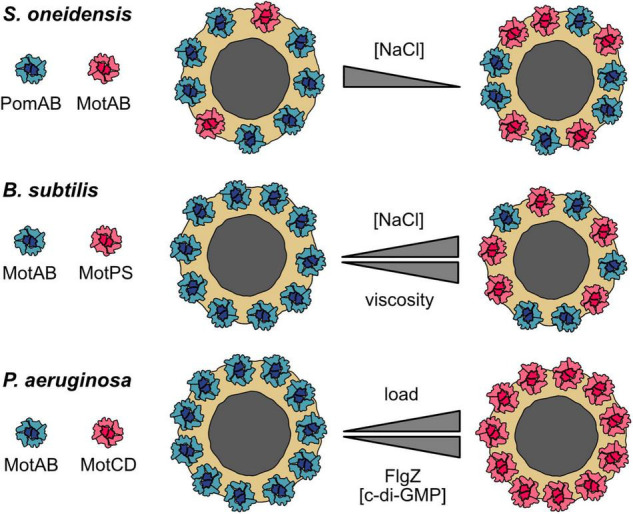
Stator dynamics and dual stator systems. Cartoons of top views of the MS-ring and C-ring (gray and yellow, respectively) with the surrounding stator units. Upper panel: *S. oneidensis* possesses two types of stator units: Na^+^-dependent PomAB (blue) and H^+^-dependent MotAB (red). A low environmental concentration of Na^+^ favors disengagement of PomAB from the motor and engagement of MotAB. At low Na^+^ concentrations, the motor runs as a hybrid, synchronously using Na^+^ and H^+^ to drive rotation. The gray triangle indicates an increasing or decreasing concentration of the coupling ion Na^+^. Middle panel: a similar motor–stator arrangement is present in *B. subtilis*, which also has H^+^-dependent MotAB (blue) and Na^+^-dependent MotPS (red) stators. The MotAB stator is dominant under most conditions, but increasing concentrations of Na^+^ and higher loads favor the incorporation of MotPS into the active stator ring. The upper triangle represents differences in the environmental Na^+^ concentration, the lower triangle increase or decrease in environmental viscosity. Lower panel: *P. aeruginosa* has two H^+^-dependent individual stator units, MotAB (blue) and MotCD (red). MotAB powers rotation during planktonic free swimming, whereas MotCD is used under conditions of high load, e.g., during swarming across surfaces (indicated by the upper gray triangle). It should be noted that it is not clear if all MotAB stators are replaced. Notably, the regulator of the flagellar motor, FlgZ, when it is binds the secondary messenger c-di-GMP, uncouples MotCD (but not MotAB) from the motor (indicated by the lower gray triangle). MotCD then interacts with the membrane-localized diguanylate cyclase SadC to stimulate production of more c-di-GMP. Thus, at high c-di-GMP concentrations, cells of *P. aeruginosa* are unlikely to induce swarming but rather remain associated tightly with the surface (see the main text for explanations).

*Shewanella oneidensis* has two types of stators to power rotation of its single polar flagellum. One of them, PomAB, is powered by Na^+^. The function of the second stator, MotAB, depends on H^+^ gradients. PomAB is the dominant stator under all conditions tested, but full swimming speed, as a measure of motor performance, at low Na^+^ concentrations requires the presence of MotAB (Paulick et al., 2009). In the absence of MotAB, about eight stator units are present in the flagellar motor, each of which is exchanged after some 30 s on average (Figure 2, upper panel). In contrast to the *Vibrio alginolyticus* motor, the PomAB stators do not uncouple from the motor, nor does the exchange half-time increase even at very low Na^+^ concentrations. This suggests that the load on the motor affects PomAB binding (Paulick et al., 2015). When MotAB was the only stator present, up to 11 stators were observed to be engaged with the motor. The rate of exchange of MotAB was similar to that of PomAB at low Na^+^ concentrations, but the rate almost doubled at high levels of Na^+^. When both stators were present, the motor exhibited a mixed configuration: In the presence of Na^+^, the ratio of PomAB to MotAB was about 7:2 with a slow turnover of PomAB. In the absence of Na^+^, the PomAB/MotAB ratio switched to 6:5, and there was a high rate of PomAB turnover (Paulick et al., 2015).

In agreement with cryo–electron tomography studies, the data indicate that, in the *S. oneidensis* motor, eleven stators can be synchronously engaged (Kaplan et al., 2019; Blagotinsek et al., 2020). In the presence of Na^+^, eight of the 11 positions are occupied by the Na^+^-dependent PomAB stator. The remaining vacant slots are taken up by MotAB stators, although these have only a meager effect on flagellar rotation. However, when the concentration of Na^+^ is low, the rate of MotAB incorporation increases and contributes significantly to motor performance (Paulick et al., 2009, 2015).

A similar motor–stator arrangement is present in *B. subtilis*, which possesses H^+^-dependent stators (MotAB) and Na^+^-conducting stators (MotPS) to power one type of flagellar motor (Ito et al., 2004, 2005). MotAB is the dominant stator for normal swimming, whereas MotPS-mediated swimming required appropriate conditions with respect to increased pH, adequate Na^+^ levels, and high viscosity as well as elevated levels of stator proteins (Ito et al., 2004, 2005; Terahara et al., 2006; Chan et al., 2014). The *B. subtilis* motor is also highly dynamic (Figure 2, middle panel): At low environmental Na^+^ concentrations, only MotAB is associated with the flagellar motor, but a mixed MotAB/MotPS motor–stator configuration occurs at elevated Na^+^ levels (Terahara et al., 2017a). In addition, increased viscosity results in a decrease in MotAB stators interacting with the flagellar motor and favors engagement of the Na^+^-dependent MotPS stators (Terahara et al., 2020). A high concentration of polysaccharides may also increase incorporation of MotPS into the motor (Terahara et al., 2017b,^2020^). It should, however, be noted that, in the wild-type *B. subtilis* strain, a significant contributing role of MotPS to swimming or swarming motility under natural conditions has yet to be demonstrated.

Both studies cited above nicely demonstrate that both *S. oneidensi*s and *B. subtilis* can adjust flagellar motor function by using more than one stator type. Notably, in both cases, hybrid motors can run synchronously on two fuels, H^+^ and Na^+^, and thus function over a wide range of Na^+^ concentrations. The motor–stator configuration can be adjusted by “in-flight” stator swapping while the motor continues to operate. The general mechanism appears to be rather simple: two distinct populations of stator precomplexes compete with each other for incorporation into the stator ring. The actual configuration is dynamically determined by the stability of the interaction of the corresponding stator with the motor, which depends on the local environmental conditions as elaborated above. In *S. oneidensis*, the motor composition is certainly governed by the Na^+^ concentration. In *B. subtilis*, the choice of stator type also depends upon the polysaccharide concentration and viscosity.

## The Two-Stator System of *Pseudomonas aeruginosa*

In the last paragraph, it was shown how bacteria may use a dynamic hybrid motor to run on two different fuels at the same time. However, there are also different examples of how different sets of stators can be used to upgrade the flagellar machinery. As the before-mentioned *S. oneidensis* and *B. subtilis*, *Pseudomonas aeruginosa* also possesses two different stator types to power rotation of the single polar flagellum (Doyle et al., 2004; Toutain et al., 2005). However, the *P. aeruginosa* system is different in that both stator units, MotAB and MotCD, are powered by H^+^ gradients. MotAB is used primarily for planktonic swimming at normal viscosity, whereas MotCD powers flagella rotation during swarming across surfaces when the load on the flagellum is high (Doyle et al., 2004; Toutain et al., 2005; Kuchma et al., 2015). Similar to *B. subtilis*, stator selection may be regulated by environmental viscosity because MotCD-motor interactions are more stable at high load (Figure 2, lower panel). Thus, the dual stator setup of *P. aeruginosa* allows a configuration better suited to motility at high load. It will be highly interesting to further study the stator exchange dynamics of this system in dependence of increasing and decreasing load. In addition, as both depend on the same coupling ion, the dual stators of *P. aeruginosa* provide an excellent model to investigate, which structural properties of the stator units allow the generation of higher torque.

Intriguingly, the *P. aeruginosa* dual stator system was additionally demonstrated to regulate and be regulated at another level: MotCD (but not MotAB) can additionally be bound by the c-di-GMP–responsive regulator FlgZ, which prevents functional rotor–stator interaction (Baker et al., 2016). Thus, a high c-di-GMP level inhibits MotCD engagement and swarming motility, and the cell rather commits to surface adhesion and biofilm formation. Notably, disengaged MotCD in the cytoplasmic membrane can make direct contact with the membrane-localized diguanylate cyclase SadC (Baker et al., 2019). This interaction stimulates c-di-GMP synthesis by SadC, which further promotes a surface-associated lifestyle. Thus, MotCD of *P. aeruginosa* not only functions as a high-duty stator for motility at elevated load but also as structural component of a feed-forward regulator that influences the decision to adopt a planktonic or surface-associated lifestyle.

## Implications and Further Questions

The studies so far have demonstrated that the dynamics underlying the interactions between the stator and the flagellar motor are used by bacteria to optimize performance of the motor according to the environmental conditions. There are, however, still numerous unsolved questions and fascinating new flagellar motor systems to be discovered.

*What is the full range of motor configurations that exist?* To date, there are three systems with multiple stators that have been characterized in detail, as elaborated above. There are, however, many more species that are likely to expand the range of possibilities for ways to regulate motor functions by stator swapping. *Aeromonas hydrophila* is among the species with multiple stators. It possesses two Na^+^-dependent stators (PomA1B1 and PomA2B2) to power its single polar flagellum (Wilhelms et al., 2009). Each stator is able to power swimming at normal and elevated viscosity. However, PomA1B1 appears to function better at lower Na^+^ concentrations. It is conceivable that *A. hydrophila* also dynamically adjusts the rotor–stator configuration in response to changes in the external Na^+^ concentration, but this remains to be shown.

An analysis of the available genome sequences indicates that some bacterial species, e.g., *Desulfovibrio* sp., not only possess two stator isoforms but also have three or even four stator units that may contribute to motor function (Thormann and Paulick, 2010). Thus, it may be hypothesized that motor systems synchronously powered by three or four types of stator units exist in nature to extend further the range over which the function of a flagellar motor may be regulated by stator switching. However, as these motor–stator systems have not yet been characterized, this possibility remains speculative for the time being.

*How is stator selection regulated?* As elaborated above, the thorough studies demonstrate that the motor–stator configuration can be passively adjusted by the stability of the interaction of one or more stator types with the motor. This regulation may depend on the concentration of the corresponding coupling ion or the load on the motor. However, it has been demonstrated that additional mechanisms to regulate stator selection exist, and we expect additional mechanisms to be identified in the future.

As the abundance of a stator unit has been shown to affect incorporation into the motor (Terahara et al., 2006), stator selection may additionally be mediated by specific upregulation or downregulation of the corresponding stator, as suggested for *A. hydrophila* (Wilhelms et al., 2009). This may be a way of affecting stator selection in other bacteria as well.

In addition, as demonstrated for *P. aeruginosa*, stators can be specifically uncoupled from the motor by binding of regulatory proteins, such as FlgZ in the presence of elevated levels of the secondary messenger c-di-GMP (Baker et al., 2016). Several such c-di-GMP–responsive regulators that may interfere with rotor–stator interaction have been described, such as MotI in *B*. *subtilis* or YcgR in *E. coli* (Boehm et al., 2010; Fang and Gomelsky, 2010; Paul et al., 2010; Gao et al., 2013; Subramanian et al., 2017; Subramanian and Kearns, 2019). Therefore, stator selection by regulator binding to the stators could occur in species with multiple stator sets. Potential regulators do not necessarily have to be c-di-GMP–binding proteins and may also be involved in other cellular processes. An example for such a protein is *B. subtilis* EpsE, a glycosyltransferase involved in synthesis of an extracellular polysaccharide. It moonlights as a clutch for the flagellar motor by binding to the flagellar C-ring (Blair et al., 2008).

*How did hybrid flagellar motors evolve?* Generally, flagellar systems with two or more stator units may have evolved either by gene duplication followed by development of functional divergence through mutations or by the genes encoding another stator system having been acquired by lateral gene transfer. Previous studies in which stators were swapped between *E. coli* and *Vibrio* sp. showed that “foreign” stators using different coupling ions can interact with the flagellar C-ring to enable motility, albeit at a significant cost in motor performance. However, in many cases, gain-of-function mutants readily emerged, indicating that even a few mutations are sufficient to improve motor–stator interaction significantly (Gosink and Häse, 2000; Asai et al., 2003; Sowa et al., 2005). In all species discussed in detail above—*S. oneidensis*, *B. subtilis*, *P. aeruginosa*, and *A. hydrophila—*the two stator systems share little identity at the level of amino acid sequence. This fact, and a significant similarity of one the stators to those from other species, suggests that, in all four cases, a second “foreign” stator has been acquired and evolved to become a helpful functional upgrade of the flagellar machinery. Notably, secondary stator systems are present in many species of *Pseudomonas*, *Bacillus*, and *Aeromonas* (Thormann and Paulick, 2010), suggesting that acquisition of the second stator occurred before phylogenetic divergence. In contrast, the MotAB stator system of *S. oneidensis* appears to be a rather recent acquisition that is present only in this particular species within the genus *Shewanella.* It may have been selected to facilitate adaptation from a marine to a fresh-water environment (Paulick et al., 2009, 2015; Brenzinger et al., 2016).

Together, the conservation of rotor–stator interactions enables functional upgrades of the flagellar motor by a later acquisition of additional stator types. The dynamics involved in stator coupling to the motor allow mixed stator configurations, even enabling the motor to use two different fuels synchronously. Motor performance can be constantly adjusted by in-flight stator swapping. So far, we have characterized a mere handful of systems, and many discoveries on flagellar systems with multiple stators remain to be discovered. The acquisition and functional adaptation of novel stators is an outstanding model system to study the mechanism of rotor–stator interaction, in particular, and the functional evolution of bacterial nanomachines, in general.

## Author Contributions

KT conceptualized and wrote the manuscript.

## Conflict of Interest

The author declares that the research was conducted in the absence of any commercial or financial relationships that could be construed as a potential conflict of interest.

## Publisher’s Note

All claims expressed in this article are solely those of the authors and do not necessarily represent those of their affiliated organizations, or those of the publisher, the editors and the reviewers. Any product that may be evaluated in this article, or claim that may be made by its manufacturer, is not guaranteed or endorsed by the publisher.
